# Forgetting What Was Where: The Fragility of Object-Location Binding

**DOI:** 10.1371/journal.pone.0048214

**Published:** 2012-10-31

**Authors:** Yoni Pertzov, Mia Yuan Dong, Muy-Cheng Peich, Masud Husain

**Affiliations:** UCL Institute of Cognitive Neuroscience and UCL Institute of Neurology, Queen Square, London, United Kingdom; University of Melbourne, Australia

## Abstract

Although we frequently take advantage of memory for objects locations in everyday life, understanding how an object’s identity is bound correctly to its location remains unclear. Here we examine how information about object identity, location and crucially object-location associations are differentially susceptible to forgetting, over variable retention intervals and memory load. In our task, participants relocated objects to their remembered locations using a touchscreen. When participants mislocalized objects, their reports were clustered around the locations of *other objects* in the array, rather than occurring randomly. These ‘swap’ errors could not be attributed to simple failure to remember either the identity or location of the objects, but rather appeared to arise from failure to bind object *identity and location* in memory. Moreover, such binding failures significantly contributed to decline in localization performance over retention time. We conclude that when objects are forgotten they do not disappear completely from memory, but rather it is the links between identity and location that are prone to be broken over time.

## Introduction

Remembering the locations of objects in space is a crucial biological function. In everyday life, gaps in visual information occur continuously (e.g. occlusions, blinks and saccades), and object location information is considered to be necessary to establish the mapping of objects visible before and after such disruptions [1]. Moreover, we often use memory of object locations for navigation [2], as well as to guide actions toward previously visible objects [3] (but see [4]). This has led to the suggestion that direction of gaze is anchored not only to the instantaneous visual scene, but also to the internal memory representation of object locations [5].

In spite of its importance, the mechanism by which object location information is maintained in memory is still unclear (for a recent review see [6]). A key question is whether object location information is integrated *with the object*, or instead held *separately*. Several studies have demonstrated that the receptive fields of neurons in higher level visual areas, which represent integrated objects, can also be sensitive to an object’s retinal position [7], [8]. However, other studies showed that these receptive fields are relatively large and tolerant to changes in object position [9]–[11], presumably leading to position invariance at the level of neural population [12], [13]. Thus, accurate memory of object location might not rely on these brain regions *alone*. Although there is evidence that memory for the identity and location of objects is sometimes multiplexed, especially in frontal and prefrontal brain regions [14], [15], the prevailing view is that memory of object location and identity is maintained in *separate* memory stores.

This conclusion arises from various sources and is consistent with several theoretical frameworks. The concept of 'object files’ [16], more recently extended to the ‘neural object file’ framework [17] proposes that spatial and temporal information allows an object representation to be maintained *without* detailed information of all its properties. Instead this ‘object file’ could act as an index [18] that points to more precise visual information which, crucially, is represented elsewhere. Results from multiple object tracking tasks reveal that participants’ recall of target location is superior to recall of its visual properties [18]–[20]. Thus, they can continuously update target locations without noticing featural changes or even identity. Such a dissociation between tracking locations and identity would not be expected if both were held together, and therefore supports the possibility that location and identity are represented separately in memory.

A second line of evidence comes from behavioural interference studies which show that while spatial memory is selectively impaired by certain tasks (e.g. movement discrimination), object memory is impaired by very different tasks (e.g. colour discrimination [21]–[23]). Further support for a dissociation between spatial and visual memory comes from reports that brain lesions can lead to deficits in spatial working memory (WM) but not to visual WM, and vice versa [22], [24], [25]. Finally, imaging studies have also reported evidence of dissociations, concluding that spatial and visual WM depend mainly on dorsal and ventral visual processing streams [26], [27] or right and left hemispheres, respectively [28]–[30].

The existence of two separate memory stores – one for object features and a different one for object location – implies that in order to remember the location of objects, the two representations have to be associated, or *bound*, somehow. Indeed recent studies have begun to characterize the specific task conditions under which identity and location information interfere with each other [31], [32]. The importance of object-location interactions is further bolstered by an intriguing proposal that location ‘pointers’ in parietal cortex are linked to visual information, and that such links might actually be necessary to establish a stable visual environment (for further description of this proposal see [33]).

Of course, extensive research has been directed to understanding how different visual features belonging to an object are bound to each other *for perception*. Many of these studies have analysed the distribution of “conjunction errors” or “illusory conjunctions” in which participants recombine features belonging to different objects erroneously, presumably reflecting a perceptual failure in feature binding [34]. However, some argue that illusory conjunctions are actually the result of post-perceptual processes [35] or acceptance of an incorrect “perceptual hypothesis” [36]. A promising avenue for research would be to study how object identity is linked to location *for memory,* by analysing the distribution of localization errors with respect to all objects in the memory array. In this case, reporting the location of the wrong object in memory (defined here as a ‘swap’ error), rather than any other random location, would be analogous to a “conjunction error” in perception.

Note that results of some previous studies might be interpreted to suggest that ‘swap’ errors should not be evident above chance levels. Thus, localization errors should mainly be spread randomly across the screen, rather than cluster around non-target items. Studies that tested memory for non-spatial visual properties have claimed that visual short term memory consists of objects with tightly bound properties [37], [38] and when objects are forgotten they are lost as a whole - leaving practically no trace behind [39], [40]. Simple extrapolation of this all-or-none view to the spatial domain might predict concurrent loss of visual and spatial information that would be reflected as random localization. However, such extrapolation should be treated with caution as position information is often considered to be a “privileged” property of objects and might therefore behave unlike other visual properties such as color and shape [41]–[43]. Surprisingly, no previous study of object-location memory has investigated the effect forgetting has on the distribution of spatial errors using an analogue measure.

Most previous reports of object location memory have, however, been concerned with other issues, such as how individual objects are remembered [44], [45], group differences in memory performance [46], [47], or whether object location is coded automatically [48], [49]. Moreover, many studies that have investigated how people recall the location of objects have used forced-choice reports (e.g. [50], [51]). Those investigations limited the report alternatives to a number of predefined locations occupied by one of the objects in the array. Such an approach does not take full advantage of analyzing the distribution of errors over space if participants are allowed to locate objects freely, without constraints, to assess whether memory failures occur randomly across space or are clustered around locations of *other objects* in the memory array. Conversely, those studies that have allowed participants to localize objects freely at any location did not report how items were localized with respect to the original locations of other objects in the memory array [52]–[54].

In our experiments, we allowed participants to freely localize objects from WM using a touch screen. In addition we analyzed errors with respect to *both* original object locations, as well as the locations of other items in the memory array. Using this approach we were able to demonstrate in experiment 1 that people often ‘swap’ the identities of objects to the locations of other items, indicative of binding failures. However, it might be argued that such failures in binding represent limitations in visual processing and not necessarily memory maintenance. To provide compelling evidence of binding failures over time in visual WM that cannot be explained by limitations in visual processing, it would be decisive to show that such errors increase with retention duration. Remarkably, none of the previous studies that have investigated WM for object location manipulated delay duration. Therefore – crucially – previous investigations could not directly address how object identity and location information is maintained, or indeed forgotten over different time intervals. Here, in experiments 2 and 3 we attempted to overcome this by assessing whether binding failures increase over time as items are held in WM.

In our first 2 experiments we used images of real complex objects. While such stimuli might be more naturalistic, they have an inherent disadvantage when studying visual memory because they are easily verbalized. Verbalization of the visual stimuli could potentially influence our results in several ways. For example, participants could memorize a list of associations (ball-right, butterfly-left) rather than rely strictly on visual memory. To investigate the robustness of our results to various types of stimuli and to decrease the effects of verbal coding (but still use complex visual objects), experiment 3 incorporated fractals rather than real objects as a stimuli.

To anticipate our results, we found that a significant number of misplaced objects were not randomly localized across space but clustered specifically at the locations of other objects in the memory array. Critically, such binding failures explain a significant amount of the localization error – forgetting – when a large number of objects have to be maintained in memory (Experiment 1) and for extended retention intervals, regardless of stimulus type (Experiments 2 & 3).

## Experiment 1

### Methods

#### Participants

Twenty-six adults (eleven female; age mean 25±5 (s.d.) years) responded to an ad published at University College London (UCL) psychology department’s subject pool or recruited from UCL Institute Of Neuroscience’s list of subjects. They all gave written informed consent to procedures approved by the local ethics committee and received compensation for their participation. All participants had normal or corrected-to-normal visual acuity by self-report.

#### Stimuli

The stimuli consisted of 60 pictures of natural objects with white background and maximum width and height of 120 pixels (∼4 degrees of visual angle) randomly selected without repetitions for every trial. Each block consisted of 50 trials including 10 trials with 1 to 5 objects. Object were presented three times in each block. Object location was determined by a Matlab script (MathWorks) in a random manner at any possible position on the screen - with several restrictions. Objects were positioned at a minimum of 4° from the edges of the screen and 7° from the centre of screen. Moreover they were never located within 10° of each other in order to prevent spatial uncertainty as a result of crowding [55] and to create a ‘sterile zone’ around the original locations of the items which is critical for the analysis of localization errors. The stimuli were presented at a viewing distance of 43 cm on an interactive touch-sensitive screen (Cintiq 18SX, Wacom) with a 1280×1024 pixel matrix, corresponding to 45.4×36.4 degrees of visual angle.

#### Procedure

Experiments were conducted in a dimly lit room. Participants sat in front of a computer screen while their head was positioned on a chinrest. Participants were allowed to freely fixate the stimuli to decrease the effect of crowding at peripheral vision [55]. A video-based tower mounted eye tracker (Eye Link1000, SR Research, Ontario, Canada) was used for recording eye movements. We used custom-built programs provided with the eye tracker for calibration and validation purposes (9 points in a random sequence) as well as for stimuli presentation and data collection. All the data analyzed here were obtained from recordings with an average absolute global validation error of less than 1 degree. Eye-tracking was discarded in 9 participants duo to technical difficulties, mainly caused by the short eye to screen distance, dictated by using touch screen reports. After each block of 50 trials there was a break which was followed by repeat calibration of the eye tracker.

**Figure 1:**
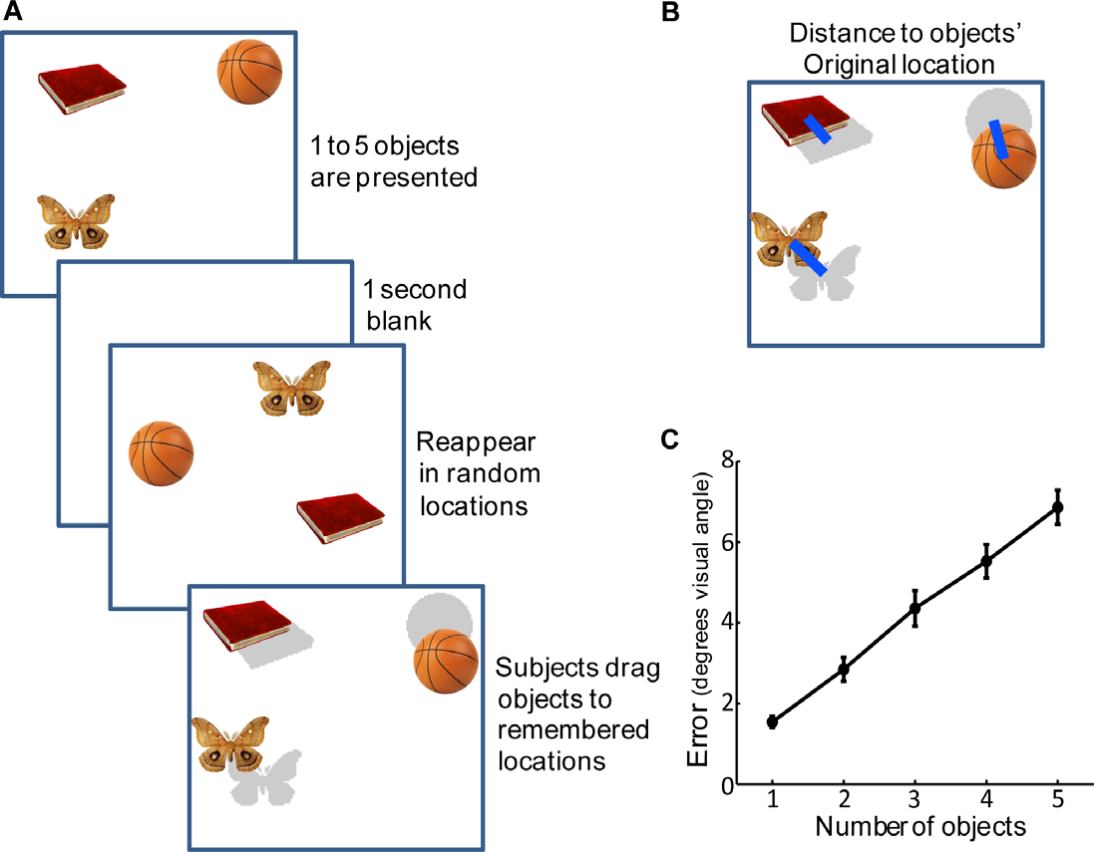
Mean localization error increases with more objects to remember. (A) Experiment 1: One to 5 *o*bjects were simultaneously presented at random locations on the screen for 1–5 seconds (1 second per item displayed). Following a delay of 1 second, the objects reappeared in novel random locations and participants were required to “drag” them using the touch screen to their remembered locations. The original locations of objects are shown here in light grey only for illustrative purposes; participants did not receive any feedback as to their errors. (B) Illustration of the dependant variable: distance between the reported locations and their matching original locations. (C) Mean localization error relative to the number of objects presented in the trial. Error bars denote SEM across participants.

The participants initiated each trial by fixating a central fixation point and pressing the space bar. The gaze position data obtained during this fixation was used to correct for slow drifts of the eye tracker. Next, an array of 1 to 5 objects was presented for 1–5 seconds respectively (to ensure there was no encoding limitation). Participants were allowed to freely view these objects and their eye position was tracked. Then a blank screen was displayed for 1 second, after which the objects reappeared in novel, random locations under the same limitations as in the initial display (Fig. 1A). Participants were required to touch and “drag” each object to its remembered location. They were free to select and move objects in any order they wished to. Participants clicked any one of the keyboard keys to confirm the objects’ locations and move to the next trial. The total duration of the experiment was 75 minutes; participants performed a practice block of 10 trials and 3 to 5 experimental blocks (150 to 250 trials).

## Results and Discussion

First we analyzed the absolute distance between the original and reported locations of the objects (Fig. 1B). Overall mean localization error increased with increasing number of objects to-be-remembered (F(4,100) = 134, p<.001, η_p_^2^ = 0.84; Fig. 1C). This relationship appeared to be linear under these experimental parameters (F(1,25) = 303, p<.001, η_p_^2^ = 0.92). Therefore additional objects recalled from working memory led to a decrease in precision of recall. Note however, that when additional objects have to be remembered and reported, the interval between the last time an object was fixated and the time its location was reported is also extended. Because the retention interval and order of fixations have been shown to affect memory performance (e.g. [53], [56]), decreased precision could be attributed to these factors rather than strictly to the number of objects in memory.

**Figure 2:**
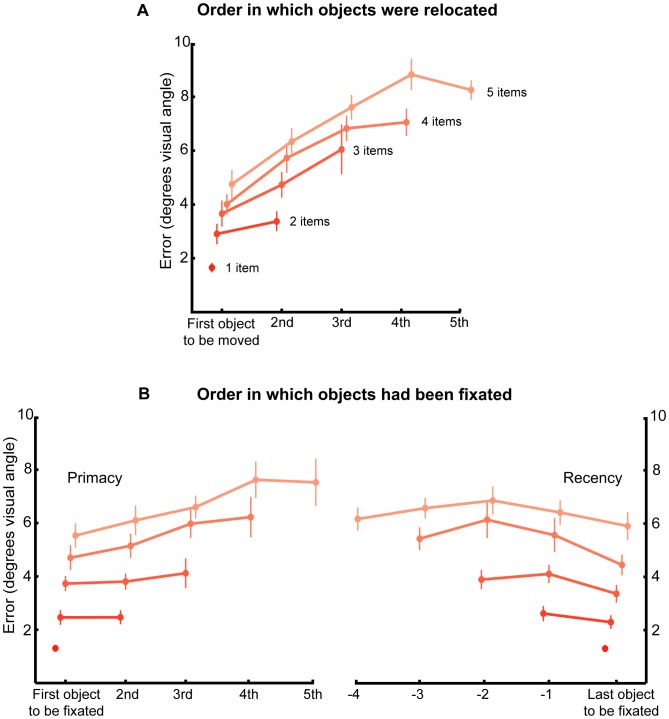
Localization error with respect to selection and fixation order. (A) Error relative to the serial order in which the objects were selected for localization. Different shades represent trials with different numbers of objects. (B) Mean localization error relative to the serial order in which the objects were fixated during the presentation period. The serial order of fixation is calculated according to the first time the object was fixated (left) and the last time it was fixated (right). Note how the error associated with either first or last object to be selected for relocation or fixated during the presentation alters systematically with total number of items in the array. Error bars denote SEM across participants.

To address this issue, we analyzed errors with respect to the sequence in which the objects were chosen to be localized (Fig. 2A). The results show two key findings. First, precision of memory worsens across the sequence in which items were localized. Second – and perhaps more importantly – even when the order in which the objects are selected is controlled for, performance worsened as the total number of objects in the array increased. For example, memory of the first object to be moved was always best, but it varied systematically with the total number of objects to be remembered (F(4,100) = 32, p<.001, η_p_^2^ = 0.56). Thus the greater the number of items in the array, the worse the absolute error, regardless of the fact that this item was the first to be moved to its remembered location. A similar pattern was also observed for the 2^nd^, 3^rd^ and 4^th^ items (Fig. 2A).

Next we considered the fact that the presence of additional items in the stimulus array means that when participants report the location of an object, on average, longer time intervals have passed since it was fixated. Thus, the decreased accuracy for additional items might not be a result of maintaining additional items in memory but rather might be strictly related to the order in which the items were fixated. To investigate this question, we calculated the error for the sequence in which the items were fixated during the initial presentation of the array. Note that in free viewing conditions objects are often refixated, so the same object could be the first *and* the last one to be fixated. Therefore we sorted the data according to the first (Fig. 2B left plot) and last time the object was fixated (Fig. 2B right plot). This analysis revealed a systematic relationship between precision of memory and both the number of items to be remembered and the order in which they were fixated.

Both a primacy and recency effects for fixation sequence order were evident: the last item to be fixated was better recalled than the averaged recall precision of all other items (2 objects: t(16) = 2.5, p = .026, Hedges’ g = 0.29; 3 objects: t(16) = 2.3, p = .033, g = 0.48; 4 objects: t(16) = 3.4, p = .004, g = 0.69; 5 objects: t(16) = 2.3, p = .038, g = 0.32). Similarly, the first item to be fixated was localized better than the other items, especially when 4 and 5 items were presented (4 objects: t(16) = 3.9, p = .002, g = 0.52; 5 objects: t(16) = 2.7, p = .016, g = 0.7). In addition, the precision of recall for the *N*th item depended upon how many other objects were in the array. Thus, for example, the error in recall of the first and last fixated object increased significantly with the number of objects in the memory (First object: F(4,64) = 49, p<.001, η_p_^2^ = 0.76; Last object: F(4,64) = 59, p<.001, η_p_^2^ = 0.79). We can conclude that the degrading effect of additional objects in memory is evident even when order of report and encoding were controlled.

**Figure 3:**
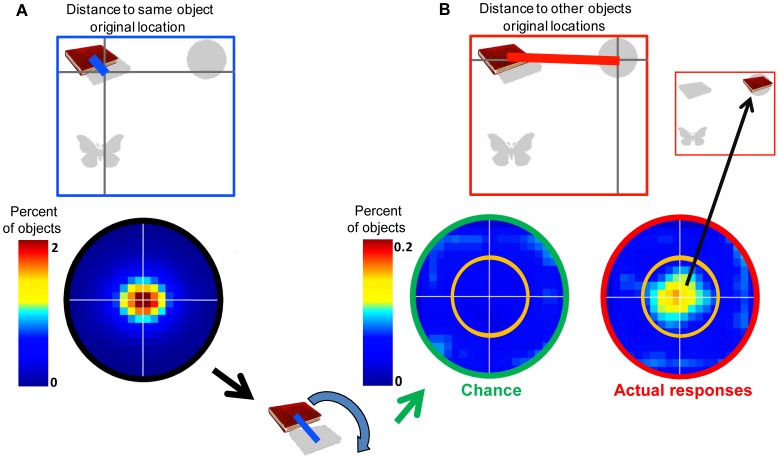
Analysis procedure for “Distance to other objects location”. (A) The vector of the error between reported and original locations can be plotted on two dimensional histograms or heat maps (radius - 10°). This shows symmetric error of memory recall around the original location of the object. (B) A similar analysis was also performed on the vector of distance between the reported location of objects and the original location of all the other objects in the trial. The rightmost heat map shows a similar error distribution, but with reduced frequency, around the original location of other objects. The heat map in the middle shows the chance level for localizing objects around the original location of other objects, computed by taking the trial’s absolute distance of error but randomizing the angular deviation from the original object location.

Next we analyzed the distribution of localization errors with respect to the *other* items in memory. For this purpose we first plotted a two-dimensional histogram or heat map, of the *vector of error* relative to the original location of the correct object, across all objects and participants. This shows that, as might be expected, errors cluster around the veridical location of an object (Fig. 3A). The symmetric distribution of errors suggests that any systematic constant error biases (often evident in localization tasks, e.g. [57], [58]) were diluted by the randomized manner the location of objects was selected.

Next we investigated how objects were localized relative to other objects in the memory array. First we plotted errors with respect to the locations of other objects, so the origin represents the true locations of *all other objects* in the display (rightmost heat map in Fig. 3B). The plot shows that although the absolute number of responses was much smaller, there was nevertheless a cluster around the locations of other objects in the memory array. Moreover, the distribution of these errors was strikingly similar to the distribution of responses around the object’s true original location (Fig. 3A).

Might this happen simply by chance? One way to calculate chance level might be to select random object reports on the screen, but this method would alter the distribution of errors around objects’ true original locations. In our analysis, we have utilized the fact that when participants localize objects particularly near the original locations of *other* objects, their responses have a specific vector of error (absolute distance *and* angular deviation) with respect to the correct location of the object. Therefore, to calculate the baseline probability of obtaining such results simply *by chance*, we kept the absolute distance of each response from the object’s original location, but randomized its angular deviation, with the proviso that the random location of the objects was within screen dimensions and the invisible margins used for generating the display.

This “random angle control” generates semi-random response patterns while preserving the distribution of error around the object original location. With this modification, only a small number of objects would be expected to be localized close to the location of other objects simply by chance, so this provides a baseline (Fig. 3 central histogram) against which to compare the actual pattern of errors (Fig. 3 right). We counted percentage of objects localized within a circumference of 5° eccentricity from the location of other objects (orange circle perimeter line on histograms). We term any errors within this perimeter as “swapped objects” or “swap errors” because they arise from swapping the location of an object with another’s.

We used a threshold of 5° because objects were never presented less than 10° from each other. Using a 5° cut-off means that the reported location of an object could never be attributed (“swapped“) to more than one object since the reported location could never be within 5° of two original locations. Because of the jitter in localization errors, using a stricter threshold (less than 5°) might lead to the erroneous exclusion of some trials in which participants reported the location of another object but in a relatively imprecise manner. In any event, using a threshold of 4° for determining “swap“ errors did not alter the qualitative nature of the results. A 5° threshold is also well above basic localization precision as measured with a single object. In addition to using this threshold, we also computed the number of errors as the percentage of objects localized between 5–10° eccentricity from other objects, i.e. not close to objects in the memory array. We also analyzed the number of objects localized within 5° of their correct original location. Such a measure might seem redundant in light of the distance of error we have previously reported (Fig. 1C) but it is important for a direct comparison with the other “thresholded” measures.

**Figure 4:**
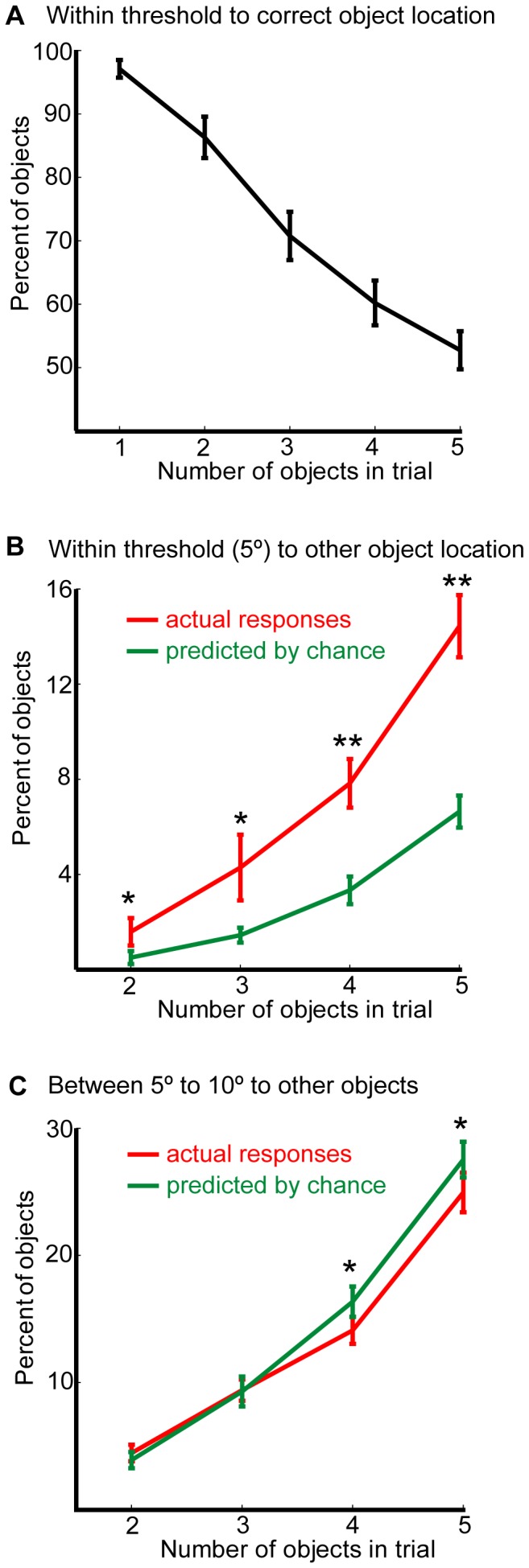
Number of ‘swapped’ objects increases with memory load. (A) Percentage of objects localized within 5° of the original location. (B) Percentage of objects localized within 5° of the original location of *other objects* from the memory array (red). Green line depicts number of objects localized near the original location of other objects expected by chance. (C) Number of objects localized to positions away (5–10°) from original location of other objects. Error bars denote SEM across participants. * p<0.05 and ** p<0.0001, for two tailed paired t tests between the actual and predicted values.

A repeated measures ANOVA with 5 set-sizes was conducted on the number of objects localized within 5° of their original location (correct localizations; Fig. 4A). Additional items to be remembered led to a decrease in the number of objects localized within 5° of original location (F(4,100) = 143, p<.001, η_p_^2^ = 0.85). The relationship between set size and within-threshold localization appeared to be linear under these experimental parameters (F(1,25) = 364, p<.001, η_p_^2^ = 0.94). Therefore additional objects recalled from working memory led to a decrease in within-threshold localizations, matching the decrease in distance or absolute error (Fig. 1C).

Was the decrease in correct localizations matched by increase in within-threshold localization around the *non-target items* (swap errors)? A 2×4 repeated-measures ANOVA with Condition (actual vs. chance) and the 4 possible number of objects (2,3,4 and 5) was conducted on percentage of swapped objects, using the 5° as a virtual perimeter for the location of an object (Fig. 4B). This analysis revealed that the number of swapped objects increased with set-size significantly more than could be predicted by chance (main effect of condition: F(1,25) = 33, p<.001, η_p_^2^ = 0.57; interaction: F(3,75) = 16, p<.001, η_p_^2^ = 0.39). Planned comparisons revealed highly significant differences between the actual and predicted values (for 2 objects: t(25) = 2.6, p = .02, g = 0.46; for 3 objects: t(25) = 2.1, p = .05, g = 0.55; for 4 objects: t(25) = 6.9, p<.0001, g = 1.05; for 5 objects: t(25) = 7.4, p<.0001, g = 1.45).

Note that the decrease in correct localizations associated with more items to-be-retained in memory (86 to 52% of objects for array sizes of 2 to 5, respectively) was greater than the increase in swap errors (2 to 14% respectively), implying that not all mislocalized objects were located around the location of other items (swap errors). Thus, some were also localized at other, presumably random, screen locations (12 to 34% of objects for array sizes of 2 to 5, respectively). Critically, however, the “random angle control” demonstrates that the number of ‘swapped’ objects could not be fully explained by simply random mislocalizations or decreased localization precision. In other words, adding additional objects to-be-remembered does indeed decrease correct within-threshold reports (Figure 4A), as well as increase both the probability of swap errors and random localizations. Importantly, by comparing the actual data to that obtained by randomizing the angle of localization errors it is possible to show that a significant proportion (8% and 14% of the objects when 4 and 5 items were presented, respectively) of actual mislocalizations were made systematically towards the direction of the original locations of other items in the array.

Conversely, ANOVA on the percentage of objects localized 5–10° from the original location of other objects (i.e. the region that is not close to their positions) reveals the opposite pattern (compare Fig. 4B to Fig. 4C). Significantly fewer objects were localized in this region than could be expected by chance alone (F(1,25) = 4.3, p<.05, η_p_^2^ = 0.15). This effect is mostly evident when 4 and 5 objects had to be remembered, confirmed by the significant interaction of condition and set-size (F(3,75) = 4.1, p = .01, η_p_^2^ = 0.14). These results are strengthened by planned t tests which indicated on significantly lower number of swaps in the actual responses when 4 and 5 objects were presented (for 2 objects: t(25) = 1.1, p = .30, g = 0.17; for 3 objects: t(25) = 0.1, p = .89, g = 0.02; for 4 objects: t(25) = −2.4, p = .02, g = −0.38; for 5 objects: t(25) = −4.9, p = .03, g = −0.34). Taken together, these analyses demonstrate that when set-size increases there is a higher probability of mislocalizing items *specifically* around the location of other objects in the memory array.

Were “swap” errors due to participants failing to remember an object’s identity and therefore mislocalizing it in one of the other remembered locations? To investigate this possibility, 16 of the participants also participated in a control experiment which was identical to the object localization experiment (Figure 1A) except that, following the delay, two objects now appeared. Participants were required to identify by touch the object they recalled had been presented earlier (the other was a foil selected from the same pool of objects). Mean performance level in the object identification task was >96% for all conditions (1–5 objects in trial). Assuming that participants also correctly identified objects strictly by chance around 4% of the trials, the upper limit on the number of objects that might be swapped because their identity was forgotten but spatial positions were not is 8% (incorrect identifications plus correct identifications by chance). This potential confound of identity failures was controlled more thoroughly in the next two experiments which specifically incorporated an object identification task into the paradigm.

## Experiment 2

**Figure 5:**
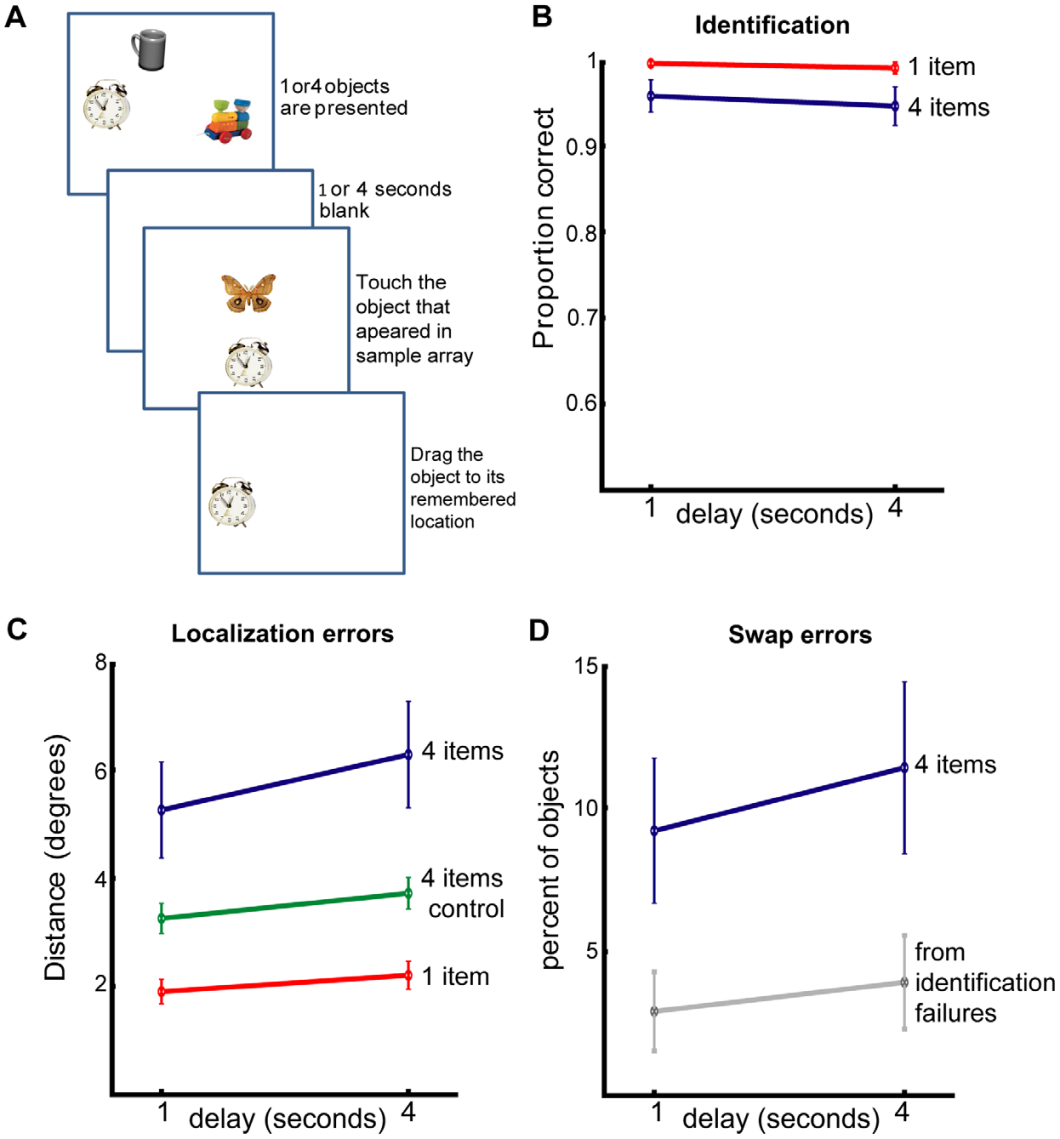
Experiment 2: Recall of object identity and object location over two different delays. (A) Experimental design: similar to experiment 1 but the delay period could be either 1 or 4 seconds, followed by a 2 alternative forced choice between one of the displayed objects and a foil. (B) Object identification performance for the different delays and number of objects in the array for real objects. For 1 object (red) and 4 objects (blue). (C) Localization errors for the different delays, including “nearest object” control (green). (D) Number of swap errors: objects localized within 5° of the original location of other objects (blue) and the number of swap errors as expected from the number of identification failures (grey). Error bars denote SEM across participants.

**Figure 6:**
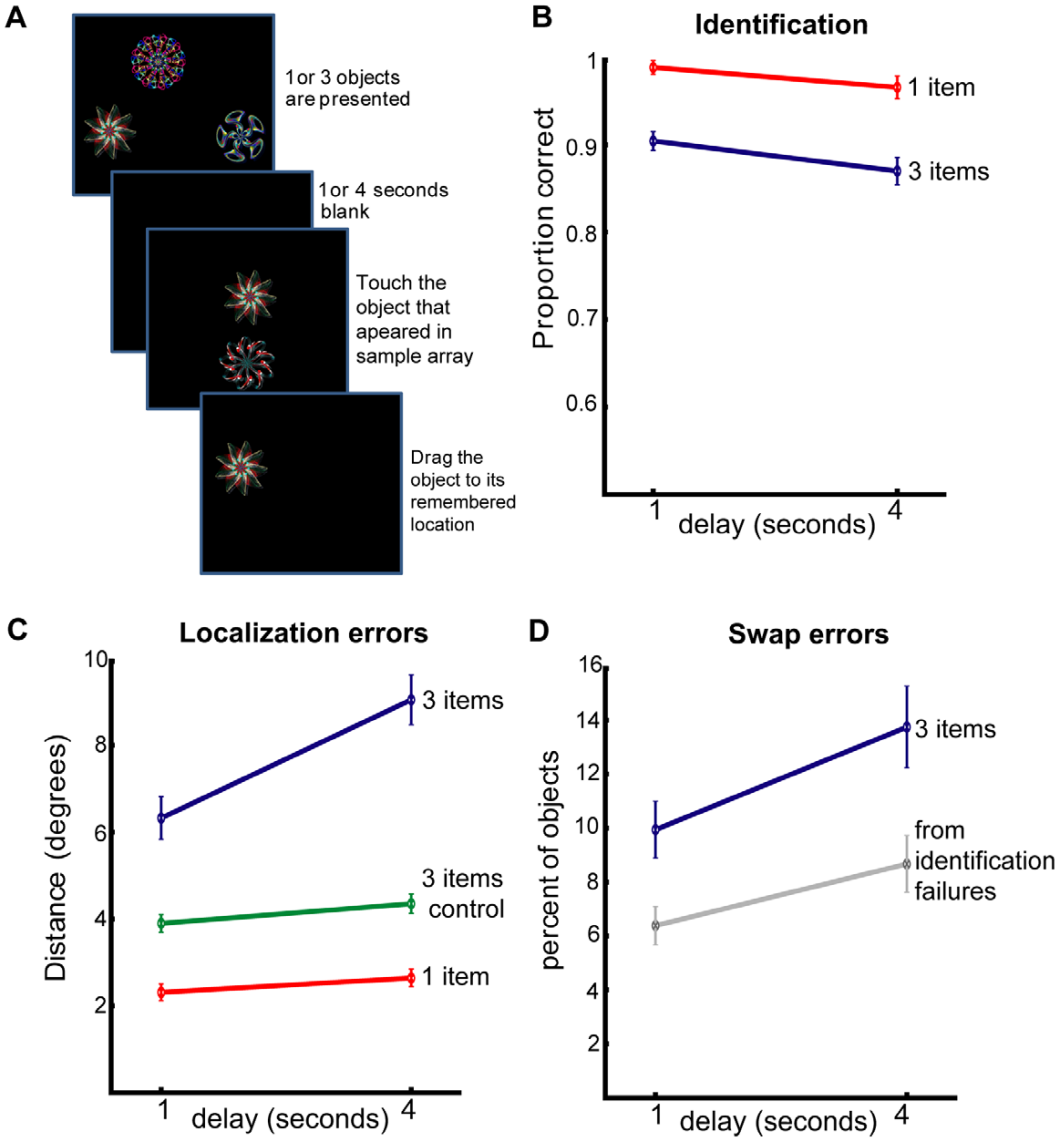
Experiment 3: Recall of identity and location of fractals over two different delays. (A) Experimental design: similar to experiment 2 but stimuli were now 3 fractals on a black background. (B) Object identification performance for the different delays for 1 (red) and 3 (blue) fractals. (C) Localization errors for the different delays. For 1 (red) and 3 (blue) fractals and “nearest object” control (green). (D) Number of objects localized within-threshold (4.5°) of the original location of other objects (blue) and the number swap errors as expected from identification failures (grey). Error bars denote SEM across participants.

Although in Experiment 1 we allowed 1 second per object to be encoded, can we be sure that swap errors relate to *memory maintenance* rather than failures related to imperfect processing at the perceptual stage? In the next two experiments we probed the effects of *retention interval* on object location memory (Figure 5A & 6A). Critically, any difference in performance between the two retention intervals could *not* be attributed to imperfect visual processing (e.g. due to visual crowding or lack of visual attention). In these experiments we also tested memory of object identity in addition to object-location memory to ensure that participants’ errors were not due to failure in recalling object identity.

### Methods

The procedure of Experiment 2 was exactly the same as that of Experiment 1, with the following exceptions.

A new sample of 12 participants was tested (four female; age mean 26±5 (s.d.) years). Each block consisted of 10 trials with 1 object and 40 trials with 4 objects. Objects repeated between 3 to 4 times in different trials within a block. The blank maintenance interval was 1 second in half of the trials and 4 seconds in the other half, in a random order. Following this, an object identification task was introduced: two objects were presented above and below central fixation. One of these objects had appeared in the memory array whereas the other one was a foil that had not. The foil object was not an unfamiliar object, but was part of the general pool of objects presented across the experiment. Participants were required to touch the remembered object and “drag” it to the remembered location. Thus in this experiment we could measure memory of object identity independently of object-location association. Localization performance was analyzed only on trials in which the objects were correctly identified.

## Results and Discussion

Identification performance was high (1 object: 99%; 4 objects 95%). A 2×2 repeated-measures ANOVA with delay (1 vs 4 seconds) and number of objects (1 or 4) was conducted on participants’ correct identifications and localization performance. The identification analysis revealed a marginal effect of number of objects (F(1,11) = 4.0, p = 0.07, η_p_^2^ = 0.27) and no significant effect of delay (F(1,11) = 2.7, p = 0.13, η_p_^2^ = 0.20) nor interaction (F(1,11) = 0.267, p = 0.62, η_p_^2^ = 0.02). This analysis suggests that participants retained the identity of objects well.

On the other hand, a similar 2×2 ANOVA of localization errors (distance between reported and true location of objects) revealed that performance worsened significantly with retention delay (main effect of delay: F(1,11) = 14.7, p = 0.003, η_p_^2^ = 0.57) as well as with the number of objects to be retained (main effect of object number: F(1,11) = 21.7, p<0.001, η_p_^2^ = 0.66). Importantly, additional objects in memory led to a significantly stronger effect of delay as revealed by a significant interaction (F(1,11) = 6.2, p = 0.03, η_p_^2^ = 0.36).

This interaction is crucial. It demonstrates that having 4 objects in memory leads to additional decrease of localization precision above that expected for memorizing only one object, over time. We further analyzed the number of swap errors for the different delays when 4 objects were memorized (swap errors could not, of course, occur when only 1 object was presented). Extending the delay by only 3 seconds significantly elevated the number of swap errors from 9 to 12% of objects (t(11) = 2.4, p = .036, g = 0.23) with a corresponding significant decrease in correct within-threshold localizations to the original target from 76 to 68% of objects (t(11) = 2.2, p = .05, g = 0.35).

Note that our analysis of the localization and swap errors consisted exclusively of trials in which participants correctly identified the object. However, some of correct identifications are expected to occur by chance. In such cases, assuming location memory is intact, participants would be expected to localize items randomly around one of the remembered locations. With four objects in the array, it would be predicted that in 25% of trials an object would be localized near its original location, while in 75% of cases it would be misassigned to non-target locations (i.e., make a swap error). We estimated the upper limit of the number of possible identity-failure swap errors by multiplying failed identification rate by 0.75 (grey line Figure 5D). The measured number of swap errors was significantly higher than the number of swaps that could be attributed simply to correct guesses of object identity (F(1,11) = 24, p<0.001, η_p_^2^ = 0.7).

To examine the role swap errors have on localization memory performance across time we calculated localization error in a slightly different manner (green line in Fig. 5C). In this control analysis, whenever an object was localized far from its original location but closer to the original location of another object, the closest location was treated *as if it was the object’s original location*. In other words, localization error was now measured as the distance between the reported location of the object and the nearest original location of any one of the other objects in the memory array. Such an analysis controls for swap errors because whenever a swap occurs it is treated as if the swapped location is the object’s original location.

We entered these controlled values, instead of the original 4 items localization errors, into an ANOVA. This manipulation showed that the interaction between number of objects and delay was no longer significant (F(1,11) = 1.3, p = 0.28, η_p_^2^ = 0.1), while both main effects prevailed (main effect of delay: F(1,11) = 20.9, p<0.001, η_p_^2^ = 0.66; main effect of object number: F(1,11) = 88.8, p<0.001, η_p_^2^ = 0.89). Thus, the additional localization error caused by the extra 3 seconds of delay and reflected in the interaction between delay and object-number, was strongly associated with swap errors.

We conclude that only 3 seconds of additional delay decreases localization performance, especially when multiple objects had to be remembered. This increased degradation when multiple items are memorized seems to be a result of an increased tendency to localize an item specifically at the location of another item from the memory array.

## Experiment 3

In the previous experiments we used images of real complex objects. While such stimuli might be more naturalistic, they have an inherent disadvantage when studying visual memory because they are easily verbalized. To decrease the effects of verbal coding but still use complex visual objects, we used fractals in the next experiment.

### Methods

The procedure was the same as that of Experiment 2, with the following exceptions. A new sample of 35 participants was tested (twenty female; age mean 34±12 (s.d.) years), each on one block of 50 trials. Stimuli consisted of 60 pictures of fractals (Sprott’s Fractal Gallery; http://sprott.physics.wisc.edu/fractals.htm) on a black background. Each fractal was presented between 2 to 3 times in different trials within the block. Following pilot experiments, in order to equate task difficulty between experiments we used 3 fractals instead of 4 real objects.

Stimuli were presented on an interactive touch-sensitive screen (Inspiron all-in-one 2320, Dell) with a 1920 × 1080 pixel matrix corresponding to 62×35 degrees of the visual angle. The changes in screen resolution and dimensions were accompanied by changes in the stimuli and location restrictions. Objects were never located within 9° of each other. They were positioned with a minimum of 3.9° from the edges of the screen and 6.5° from the centre of screen. Threshold for swap errors was therefore 4.5°. Eye tracking was not performed and chinrest was not used in this experiment. Identification, localization and number of within-threshold localizations were analysed by a 2×2 repeated-measures ANOVA with delay (1 vs 4 seconds) and number of items (1 or 3 fractals).

## Results and Discussion

Because this experiment was different to the previous one with respect to several factors, e.g. number and type of stimuli as well as screen size, we do not make any quantitative comparisons between the two. Instead, we concentrate only on *qualitative* differences and similarities.

Identification performance was again very good (98% for 1 fractal and 89% for 3 fractals). ANOVA on identification performance revealed a significant effect of number of objects (F(1,34) = 48, p<0.001, η_p_^2^ = 0.59) and of delay (F(1,34) = 6, p = 0.02, η_p_^2^ = 0.16), but an insignificant interaction between object-number and delay (F(1,34) = 0.3, p = 0.59, η_p_^2^ = 0.01). Note that in Experiment 2 identification performance was not significantly influenced by delay. However, the similar effect size (η_p_^2^ of 0.2 and 0.16 in experiment 2 and 3, respectively) suggests that this difference is a result of the greater statistical power in Experiment 3 due to a larger number of participants.

Similar to identity information, *localization performance* also revealed significant main effects of delay (F(1,34) = 62, p<0.001, η_p_^2^ = 0.65) and object number (F(1,34) = 196, p<0.001, η_p_^2^ = 0.85). However, unlike to identification performance the interaction between the two was highly significance (F(1,34) = 55, p<0.001, η_p_^2^ = 0.62). These results replicate our previous findings that additional objects in memory lead to steeper degradation of localization performance. Longer retention intervals are again associated with decreased number of correct within-threshold localizations (from 57 to 48%, t(34) = 3.6, p = .001, g = 0.44) as well as an increased number of swap errors (from 10 to 14% of objects; t(34) = 2.3, p = .028, g = 0.49).

Similarly to experiment 2, when we controlled localization performance for swap errors by analyzing the distance between the reported location of the object and the nearest original location of *any* other object (green line in Fig. 6C), the interaction between the number of objects and delay was completely abolished (F(1,34) = 0.3, p = 0.56, η_p_^2^ = 0.01), but both main effects prevailed (delay: F(1,34) = 11, p = 0.002, η_p_^2^ = 0.25; object number: F(1,34) = 120, p<0.001, η_p_^2^ = 0.80). Thus, these results further strengthen the conclusion that increasing localization error with time due to having multiple objects to remember (as manifested in the interaction between object-number and delay) is largely due to swap errors.

Note that only trials in which objects were correctly identified were entered into the analysis. Therefore swapped objects are unlikely to be a result of failure to remember object identity. Nevertheless, some of the correct identifications are expected to occur by chance, and assuming location memory is intact, participants are expected to localize the items randomly in one of the remembered locations. In one out of three of such cases the object will be localized near its original location and in the remaining two thirds, around the other objects.

Similarly to the analysis in Experiment 2, we estimated the upper limit on the number of swap errors that could be attributed to guessing the correct identity by multiplying the percentages of the failed identification with two thirds (grey line Figure 6D). To compare the measured number of swap errors to the expected one we performed a 2×2 repeated-measures ANOVA with type (measured vs expected) and delay (1 vs 4 seconds) as within participant factors. The number of swap errors was significantly higher than could be expected using identification errors (F(1,34) = 32, p<0.001, η_p_^2^ = 0.49). We conclude that despite the various differences in the experimental settings (e.g. stimuli and screen size) this experiment replicated the qualitative nature of the results of experiment 2. This provides important support for the conclusions that only 3 seconds of additional delay decrease localization performance, especially when several objects have to be remembered. This degradation is associated with an increased probability to localize an item specifically at the location of another item in the memory array.

## Discussion

In our experiments, participants were required to re-locate objects to their remembered locations. More objects in memory, as well as longer retention intervals, led to a progressive increase in mean localization error. Our analysis suggests that this additional error was caused by mislocalization to the locations of *other objects* in the memory array, presumably due to the fragility of links between object identity and location.

Such errors cannot be due simply to a failure to remember the identity of objects because memory for identity was very good and produced far fewer errors than the number of ‘swap errors’. Indeed, only trials with correct identification were actually included in our analysis. Furthermore, the number of swap errors significantly exceeded the higher limit on swap errors that could be expected from correct identifications by chance. So such errors cannot be explained by simply forgetting object identity, but neither can they be explained by simple degradation in spatial memory as object locations (regardless of their identity) were clearly remembered well (see figure 2B and “nearest object” controls). Thus, we conclude that swap errors are most likely to arise from binding failure between object identity and location information.

It has been shown before that locating objects to their correct locations is more difficult than remembering their identity or locations alone [50], [54], suggesting that links between object identity and location are particularly fragile. However, these studies did not investigate the distribution of errors or,more importantly, did not manipulate delay duration. Therefore those studies could not directly address how object identity and location information is maintained, or indeed forgotten over different time intervals. We found that extending the maintenance period by only 3 seconds led to significantly higher number of binding failures supporting the claim that resources are indeed required to bind visual features to locations in memory [59] and challenging the claim that objects are maintained as an integrated unit in memory and forgotten as entirety [40].

The effect of retention interval on binding failures have direct implications for the “episodic buffer”, the recent addition to the classic multicomponent model of working memory proposed by Baddeley and Hitch [60]. The episodic buffer is assumed to be a limited capacity storage system capable of holding bound objects, but not performing the binding [61]. Importantly, in our experiment, participants could not predict the delay duration at the time the memory array was presented so presumably visual processing and feature integration were identical in both delay conditions. Thus the elevated number of binding failures cannot be attributed to perceptual failures and must be prejudiced by the time over which the objects’ representations were maintained in memory, i.e. in the episodic buffer. These findings shed light on the limitations that the “episodic buffer” have: identity and location information, potentially held in different brain regions [22], [24], [25], are not necessarily kept tightly bound in episodic buffer. They need to be actively linked over time for veridical recall of which object was where. We make no claim here as to the manner in which locations are memorized on their own. Locations could be represented either relative to the current fixation point [62], to each other [63] or relative to the scene and context [44], [45], [64].

Previous studies have investigated the role of location in short-term memory of object identity. In change detection tasks, at short delays, changing the location of objects between stimuli and test impairs detection of change [63], suggesting a close link between objects and their locations in memory. Interestingly, the effect of scrambling item locations was found to diminish at longer delays [65]. This finding would be consistent with our conclusion that the links between identity and location degrade with time when *multiple items* have to be remembered.

The swap errors or object-location binding failures discussed here have a clear similarity to the phenomenon of “illusory conjunctions” reported in the attention literature which was suggested to demonstrate “misbinding” of different visual features [34]. Treisman and colleagues have demonstrated that when participants are presented with several objects and later required to report the different features belonging to one of them, they often made a specific kind of error, often called a conjunction error. Rather than erroneously reporting a random value, they often swap features belonging to different objects. Such errors were suggested to be a result of insufficient attentional resources that are needed, according to the Feature Integration Theory (FIT), to bind together distinct features.

However, there have been alternative interpretations. One study reported that the frequency of feature binding errors across various conditions is better explained by uncertainty about the location of visual features than FIT [66]. While another investigation argued that the attention manipulation in the original Treisman & Schmidt study was confounded by several factors. A more controlled manipulation of attention, in their view, reveals that the availability of attention resources does not in fact influence the frequency of conjunction errors [35]. Instead, they argued that conjunction errors are affected by post-perceptual rather than perceptual processes.

Our findings demonstrate specifically that degradation of information during maintenance in WM, often closely linked to attention, can contribute to apparent binding failures at the report stage. The additional swap errors we report for longer retention intervals are clearly a result of post perceptual processes that relate to increasing uncertainty about the location of the objects in a highly specific manner: being biased towards the locations of other objects in the memory array.

This study complements and extends recent findings from a color matching task [67], in which, following a brief delay, participants had to reproduce the color of an oriented bar from a prior array of several bars of different colors and orientations. Bays, Catalano and Husain (2009) found that a significant amount of the variability in the response could be explained by mis-reporting the features of the wrong item in memory. Moreover, such errors greatly increased when additional items had to be remembered. Most interestingly, another study demonstrated that errors resulting from visual crowding are not purely random, but they are similarly biased towards the features of the distractor items [68]. Here we demonstrated that similar misreporting, or ‘swap’ errors, also have a critical role in the increased *localization* errors resulting from additional items in memory, as well as extended retention intervals.

To conclude, in support of the existence of distinct memory representation for location and identity of objects, we have found that extending the retention interval by only 3 seconds led to an increased probability to swap the correct location and identity of objects held in memory, in a manner that could not be explained by forgetting of object identity or location alone. Such binding failures significantly contribute to rapid short-term forgetting as measured by the decline in localization performance across time. Thus, when objects are forgotten they do not disappear completely from memory, as previously claimed, but rather the links to their locations are gradually broken.
